# Design and Optimization of a High-Performance Self-Powered MXene–Perovskite Type-II Heterojunction Photodetector

**DOI:** 10.3390/mi17101130

**Published:** 2026-09-28

**Authors:** Amal M. Al-Amri

**Affiliations:** Physics Department, College of Science & Arts, King Abdulaziz University, Rabigh 25724, Saudi Arabia; amsalamri@kau.edu.sa

**Keywords:** proviskite, heterojunction, photodetectors, self-powered

## Abstract

The development of high-performance, self-powered, and lead-free photodetectors has attracted significant attention for next-generation optoelectronic applications. In this work, a novel Mo_2_TiC_2_/CsSnI_3_ staggered (type-II) heterojunction photodetector (MPH-PD) is designed, optimized, and simulated using COMSOL Multiphysics. The device consists of an FTO/Mo_2_TiC_2_/CsSnI_3_/Au architecture that demonstrates excellent optical confinement, strong visible-light absorption with quantum efficiency above 80%, favorable conduction- and valence-band offsets of 0.30 eV and 0.25 eV, efficient charge separation, and suppressed recombination. Under AM1.5G illumination, the optimized device exhibits a very high light-to-dark current ratio of 10^8^ under reverse-bias conditions and 10^13^ at zero bias. The photodetector achieves an excellent responsivity of 0.38 A/W, a peak detectivity of 2.12 × 10^15^ Jones, and a minimum noise equivalent power of about 4.7 × 10^−16^ WHz^−1/2^ at a 550 nm wavelength of the incident spectrum. Furthermore, the temperature-dependent simulation revealed optimum performance near room temperature with maximized responsivity, and detectivity with minimized noise equivalent power. Based on these results, the proposed MPH-PD demonstrates its significant potential for lead-free, self-powered photodetection technologies and provides a promising approach for further experimental research.

## 1. Introduction

The widespread application of photodetectors across different scientific and industrial fields is due to their significant contributions to improving quality of life through advancing medicine, science, technology, and communication [1,2]. There are certain key parameters that determine the performance of a photodetector in terms of different qualities of the device. One of the most important parameters is responsivity (R), which measures the efficiency of a photodetector in converting the optical power into an electrical signal. Another important parameter in photodetector operation is dark current. High dark current can considerably reduce the sensitivity of the device due to the poor signal-to-noise ratio. Detectivity (D) indicates the sensitivity of a photodetector to weak and low-intensity light signals and is a crucial factor for determining the performance of the detector [3,4,5]. Most present commercial photodetectors are composed of silicon-based semiconductors, gallium phosphide (GaP), and lead sulfide (PbS). The complex and costly production methods required for these devices are a major obstacle to their further development. Polymer and quantum dot photodetectors (QDPs) offer a cost-effective alternative that can be tuned to detect light signals in a spectral range from visible to near-infrared wavelengths [6]. However, these technologies are constrained by their relatively poor light absorption and inferior electrical characteristics, which limit their suitability for the intended application [7]. Conventional photodetectors, such as GaAs and GaN, are recognized for their high sensitivity. The responsivity of a GaAs photodetector at 1000 nm is 0.35 A/W. Such devices are incredibly sensitive to near-infrared radiation above 900 nm; however, they exhibit limited efficacy in detecting visible or ultraviolet radiation [8]. This factor has limited their use in broadband photodetectors. Photodetectors made with gallium arsenide (GaAs) are sensitive to temperature changes, which affect their performance [9]. To address these issues, researchers have explored alternative materials with properties that are well suited for photodetector applications.

Perovskite materials have shown great potential in optoelectronic applications, including photodetection. Their excellent properties, namely, low manufacturing cost, tunable bandgap, high sensitivity, and solution processing, make these materials highly desirable in the field of optoelectronics technology [10,11,12,13]. Moreover, perovskites have remarkable characteristics that make them ideally suited for use in various optoelectronic devices such as solar cells, optical pump lasers, light-emitting diodes, and photoelectric detectors. CsSnI_3_ represents a non-toxic alternative to lead-based perovskite materials [14,15]. It is a low-cost material with a narrow energy band gap (1.3 eV), high light-harvesting efficiency, and outstanding thermal stability, making it an ideal choice for sustainable perovskite optoelectronics [16,17]. MXenes are represented by the general formula M_n+1_X_n_T_X_ (where M is an early transition metal, X is carbon and/or nitrogen, T represents surface terminations, and n ranges from 1 to 4) and possess metallic conductivity higher than 10,000 S/cm, a hydrophilic surface suitable for solution processing, and tunable optical properties through surface chemistry manipulation [18]. Ti_3_C_2_T_x_ MXene exhibits significant light absorption from the visible to near-infrared region and is therefore suitable for optoelectronic applications [19]. The low-temperature process and cost-effectiveness of MXene fabrication result in improved homogeneity, yield, and throughput, thus facilitating improved scalability. In a recent study, an InP-based side-illuminated Schottky photodetector (SIMS-PD) was integrated into an InP substrate and achieved a responsivity of 2.31 A/W at 1.55 μm, ultra-low dark current of 5 × 10^−16^ A, and excellent detectivity of 2.1 × 10^13^ Jones, with a bandwidth of 42 GHz. The MXene exhibits improved properties owing to its high metallic conductivity of 8500 S/cm, tunable work function of 4.1 to 4.8 eV, and large optical absorption coefficient of 3.8 × 10^5^ cm^−1^. The field confinement at the MXene–InP interface has been enhanced with the proposed device architecture, with an 85% modal overlap and 69% quantum efficiency [20].

Despite the aforementioned benefits, little research has been conducted on the potential of an MXene/perovskite heterojunction (MPH) for enhanced photodetection. Its small bandgap, high optical absorption, and good charge transport properties make CsSnI_3_ a promising lead-free perovskite for broadband photodetection applications. However, its photodetection performance can be limited by carrier recombination and suboptimal charge extraction. Mo_2_TiC_2_ has unparalleled electrical conductivity, high carrier mobility, and tunable electronic characteristics, which enable rapid carrier transport and improve charge collection at interfaces. An MPH can improve photodetection performance by promoting efficient separation of photocarriers, which is facilitated by good band alignment, a built-in internal electric field, and an interface electric field. Furthermore, Mo_2_TiC_2_ is thermally and environmentally stable under various conditions, potentially ensuring the reliability of device operation under different environmental conditions. The optoelectronic properties, transport dynamics of electronic carriers, thickness optimization, and temperature dependence of the response of MXene/perovskite heterojunction photodetectors are currently unknown. In this study, a unique FTO/Mo_2_TiC_2_/CsSnI_3_/Au heterojunction photodetector is comprehensively analyzed using COMSOL Multiphysics software 6.4. The aim of this study is to provide fundamental insights into the charge transport mechanisms and establish a reliable foundation for the development of future self-powered photodetection applications.

## 2. Computational and Structural Details

COMSOL Multiphysics was used for the design and simulation of the Mo_2_TiC_2_/CsSnI_3_ heterojunction photodetector (MPH-PD). A detailed list of the different reported parameters for Mo_2_TiC_2_ and CsSnI_3_ is provided in Table 1. COMSOL Multiphysics was used to optimize the structure for different thicknesses for both layer, the solar spectrum and temperature. The continuity equations, drift–diffusion equations, Poisson equations, and photogeneration equation are solved numerically using the finite element method (FEM) and suitable boundary conditions in COMSOL Multiphysics. This advanced computational tool allows researchers to closely examine a variety of geometric and physical features of their models and to then focus their investigation on a particular design consideration [21,22,23]. Carrier recombination was modeled by the built-in Shockley–Read–Hall (SRH) recombination model, along with the built-in trap-assisted surface recombination formulation. The fully passivated surface had an assigned surface recombination velocity (SRV) of 0.001 m s^−1^, and the partially passivated surface was given an SRV between 10 and 100 m s^−1^. The computational domain was discretized with a mesh sufficiently fine near the surfaces to capture the carrier-density variations due to surface recombination. The steady-state carrier-density distribution was first calculated in COMSOL’s stationary solver for the conditions of generation. The steady-state solution was then used as initial conditions for a time-dependent study, where the generation term was turned off to mimic the loss of carriers from the system due to the removal of the excitation source. The total carrier density inside of the computational domain was calculated at each time step, and the effective carrier lifetime was calculated from the time it takes for the integrated carrier density to drop down to 1/e of its steady-state value as listed in Table 1.

In this work, the idealized pristine termination-free form of Mo_2_TiC_2_ is taken into consideration. The electronic and transport parameters in the COMSOL model are device-level representative parameters and taken from the numerical literature [24]. The band gap (1.35 eV) is in particular the effective semiconductor parameter used in the device simulation. This approximation is used to facilitate the modeling of carrier transport in the MXene layer by drift–diffusion methods. It is important to note that the electronic structure of the experimentally synthesized Mo_2_TiC_2_T_x_ is very sensitive to the surface termination, and that O-, OH-, and F-terminated systems can have significantly different band structures, surface work functions and properties of their carriers.

**Table 1:** Various reported parameters associated with Mo_2_TiC_2_ and CsSnI_3_ used in the simulation.

Layer Properties	CsSnI_3_ [25]	Mo_2_TiC_2_ [24]
Thickness (nm)	500 (Optimized)	350 (Optimized)
Band gap (eV)	1.30	1.35
Electron affinity (eV)	3.90 [26]	3.6
Dielectric permittivity (relative)	9.93	10
Electron thermal velocity (cm/s)	1 × 10^7^	1 × 10^7^
Hole thermal velocity (cm/s)	1 × 10^7^	1 × 10^7^
CB effective DOS (cm^−3^)	1.0 × 10^19^	2.2 × 10^18^
VB effective DOS (cm^−3^)	1.0 × 10^18^	1.8 × 10^19^
Donor density Nᴅ (cm^−3^)	0	1.0 × 10^16^
Acceptor density Nᴀ (cm^−3^)	1 × 10^15^	0
Electron Mobility (cm^2^/Vs)	1500	40
Hole mobility (cm^2^/Vs)	585	10
Bulk defect density (cm^−3^)	1 × 10^12^	1 × 10^12^
Surface recombination velocity SRV (ms^−1^)	0.001	100
Capture cross for electrons/holes σ (cm^2^)	10^−18^/10^−17^	10^−18^/10^−17^

The proposed MPH-PD is presented in Figure 1a, which consists of a transparent thin layer of fluorine-doped tin oxide (FTO) as the front electrode and gold (Au) as the back contact, with work functions of 4.04 eV and 5.3 eV, respectively [27]. The layered structure is meticulously designed to maximize optical absorption and provide efficient charge separation and optimum charge transport in the active region. The photodetection region consists of a heterojunction between Mo_2_TiC_2_ and CsSnI_3_ perovskite absorbers. The Mo_2_TiC_2_ layer is 150–350 nm thick and is exposed to light. The CsSnI_3_ perovskite is placed next to the MXene layer and has a high optical absorption coefficient, a suitable band gap, and good carrier transport properties; it is therefore used as the main photoactive material. When light is shone on the perovskite material, the photons are absorbed by the semiconductor and electron–hole pairs are created. These pairs are then subsequently separated in the electric field present at the MPH interface. The counter electrode comprises a gold back contact with high collecting efficiency and effective carrier extraction capacity. The proposed MPH exhibits an energy band gap discontinuity at the conduction band minimum (Ec) and valence band maximum (Ev), as shown in Figure 1b. The band alignment was determined as per Equations (1) and (2) [28]

(1) $$∆E_{c}=\chi_{peroveskite}-\chi_{MXene}$$

(2) $$∆E_{v}={(E}_{g(perovskite)}-E_{g(MXene)})+∆E_{c}$$

where χ and E_g_ denote the electron affinity and energy band gap, respectively. The positive values of ∆Ec (0.30 eV) and ∆Ev (0.25 eV) indicate that the conduction band edge of CsSnI_3_ is at a higher energy level than that of Mo_2_TiC_2_, and the valence band edge of CsSnI_3_ is also at a higher energy level than that of Mo_2_TiC_2_, confirming the formation of a staggered type-II heterojunction [29]. For the type-II band configuration, the electrons tend to transfer from the CsSnI_3_ absorber layer to the Mo_2_TiC_2_ layer because of the low ∆Ec, and the holes are retained in the CsSnI_3_ absorber layer because of the high ∆Ev. Furthermore, the charge redistribution at the interface results in bending of the energy bands and formation of a built-in electric field in the depletion region. The built-in electric field enables efficient separation and transport of electron–hole pairs under optical illumination without requiring an external bias voltage. The MPH not only possesses a high charge separation efficiency but also has low charge recombination and excellent carrier transport, making it well suited for high-performance self-powered photodetectors. The atomic configuration of the MXene layer of Mo_2_TiC_2_ is presented in Figure 1c. It consists of a 2D framework containing transition-metal atoms of molybdenum and titanium linked by carbon atoms [30]. The finite-element mesh used for the COMSOL Multiphysics simulations is presented in Figure 1d. The finite-element mesh was refined around the CsSnI_3_/Mo_2_TiC_2_ heterojunction to resolve the large spatial variations in the electrostatic potential [Figure 2a], carrier concentration [Figure 3d] and electric field [Figure 4d]. In areas where large carrier-density gradients occurred, additional local refinement was applied. The final refined mesh [Figure 1d] was used in all subsequent optical, carrier-transport and device-performance calculations. To verify the numerical solution, the final mesh gave an optimized photocurrent, responsivity, dark-current density, and specific detectivity at a carrier density. The dark current density was measured in particular because of its dominating influence on the computed detectivity. The same sophisticated mesh was used for all subsequent thickness, carrier-density, defect-density, and optoelectronic performance simulations. To resolve spatial variations in carrier concentration, electric potential, electric field distribution, and photogeneration rate within the heterojunction structure, the computational domain was meshed with a dense triangular mesh. The mesh density is intentionally increased near the MPH interface, where the electric-field gradients and band bending are the largest. The increased mesh density in this region enhances numerical accuracy and facilitates convergence of the coupled semiconductor–electromagnetic equations. Regions located away from the interface, however, have relatively small spatial variations and do not require the same degree of mesh refinement. This refinement reduction lowers computational costs while maintaining solution accuracy.

## 3. Electric Field Distribution

The spatial distribution of the electric field norm at the surface of the MPH-PD, for an excitation frequency of 333.1 THz, is presented in Figure 2a. The electric field strength is not uniform throughout the thickness of the device; rather, it varies from 25 to 62 Vm^−1^. The results indicate that the electric field intensity is enhanced near the MPH interface, which clearly demonstrates strong electromagnetic confinement and interaction of the optical wave with the active layers [31]. The electric field distribution determines the rate of photogeneration and charge carrier separation inside the device. Maxwell’s equations demonstrate that the local optical generation is proportional to the absorbed electromagnetic power density, which depends on the electric field intensity as given by Equation (3) [32]

(3) $$P_{abs}=\frac{1}{2}\omega \epsilon_{o}\epsilon^{\prime \prime }|E^{2}|\;$$

where ω is the angular frequency of incident light, $\epsilon_{o}$ is the vacuum permittivity, $\epsilon^{\prime \prime }$ is the imaginary component of the material’s relative permittivity, and $\left|E^{2}\right|$ is the squared magnitude of the local electric field. As a result, the region with higher electric field intensity will facilitate more effective photon absorption and electron–hole pair generation. The periodic electric field variation as a function of the thickness of the device suggests the formation of standing-wave-type optical modes due to multiple reflections and interference in the multilayer structure. Thus, the electric field profile confirms that the proposed heterostructure offers desirable optical confinement and extraction properties, which are crucial to obtain high responsivity and self-powered photodetection performance. To assess the robustness of the proposed heterojunction, the carrier densities of the CsSnI_3_ absorber layer and the MXene Mo_2_TiC_2_ layer were also varied simultaneously between 10^13^ and 10^18^ cm^−3^ as shown in Figure 2b. The analysis shows that the optimum device response is possible at around 10^15^ cm^−3^ for CsSnI_3_ and 10^16^ cm^−3^ for Mo_2_TiC_2_. The effects of carrier density were further explored using the generation and recombination rates as shown in Figure 2c. The generation rate does not depend on the absorber carrier density (mainly determined by the incident optical field and light absorption), while the recombination rate increases with increasing carrier density of CsSnI_3_ (increased chance of carrier–carrier and trap-mediated recombination). The recombination rate in the Mo_2_TiC_2_ layer, on the other hand, decreases with increasing absorber carrier density, which can be explained by the increased carrier transfer/extraction from the perovskite to the Mo_2_TiC_2_ layer resulting in decreased carrier density in the Mo_2_TiC_2_ layer for recombination [33,34].

The bulk defect density (N_t_) of the CsSnI_3_ and Mo_2_TiC_2_ layers was also studied in the range 10^10^ cm^−3^–10^15^ cm^−3^. The results indicate that the defect density in the CsSnI_3_ absorber can have a significant impact on responsivity compared to the MXene layer. The responsivity decreases slightly with increasing density of absorber defects up to approximately 10^14^ cm^−3^, then drops drastically at higher defect densities up to 10^18^ cm^−3^, because of the increasing number of trap-assisted recombination centers [35,36,37]. The variation in the Mo_2_TiC_2_ defect density, on the other hand, causes only a relatively small change in responsivity in the range investigated. The results suggest that the defect density of the CsSnI_3_ absorber is also particularly significant for achieving efficient carrier collection and high responsivity of the photodetectors.

## 4. Optical Response

To understand optical confinement, such as photon-harvesting capability, charge-generation efficiency, and the behavior of charge transport, we investigated the absorbance, photogeneration rate, transmittance, and reflectance in addition to the distribution of the carrier density of the proposed MPH-PD, as depicted in Figure 3. The wide absorbance response of MPH in the wavelength range from 300 to 900 nm is presented in Figure 3a. It exhibits a moderate response in the UV range below 400 nm, with values consistently in the range of 0.09. The absorbance increases gradually and achieves a peak value of 0.52 at 720 nm. Due to the high optical absorption coefficient of CsSnI_3,_ in addition to the excellent light-trapping and metallic optical response of the Mo_2_TiC_2_ layer, MPH exhibits a superior response in the visible spectrum with its wide absorption plateau [38,39]. The localization of the EM field near the heterojunction interface leads to a higher EM field intensity in this region and thus higher photon absorption. The absorbance decreases rapidly when the photon energy approaches the bandgap of CsSnI_3_ beyond 750 nm. At longer wavelengths, the rhythmic behavior results from interference effects and standing-wave optical modes. The generation rate shown in Figure 3b starts increasing from 300 nm and peaks at 8.0 × 10^5^ s^−1^ in the range of 500 to 700 nm of the visible spectrum. This behavior follows the absorbance response and is consistent with the electromagnetic absorption theory that the optical generation rate is directly proportional to the absorbed power density and local electric field intensity [40]. For wavelengths beyond 700 nm, the generation rate drops quickly because the photon energy approaches the absorber bandgap, limiting the inter-band transition probability. The elevated generation rate in the visible spectrum directly affects the internal quantum efficiency and photocurrent output. The transmission spectrum shown in Figure 3c is negligible over the ultraviolet and visible range (300–720 nm), indicating that almost all of the photons are either absorbed or reflected back. The low transmission recorded throughout a large portion of the working spectral range suggests high optical confinement and efficient use of the incident photons in the working active layers. Transmission starts to rise at wavelengths greater than 730 nm and peaks at 0.11 at 790 nm. The transmission increases at long wavelengths because the energy of the photons is not sufficient to be absorbed effectively by the CsSnI_3_ absorber.

The reflectance is calculated using the relation (A + T + R = 1) as shown in Figure 3e. The overall spectra thus show a coherent account of the optical power in the incident beam and suggest that the unabsorbed optical power is mostly due to reflections. The findings confirm that this device possesses significant broadband optical absorption in CsSnI_3_, without the need to make the assumption of complete photon harvesting.

Figure 3d presents the equilibrium carrier density distribution profile of the device. The heterointerface at 500 nm exhibits a significant electron/hole redistribution due to the strong built-in electric field. The region of CsSnI_3_ exhibits a high hole concentration, roughly 10^18^ to 10^19^ cm^−3^, and the electron concentration is low. The electron density in the Mo_2_TiC_2_ layer is significantly higher (10^18^ cm^−3^), whereas the hole concentration is lower compared to the CsSnI_3_ layer. The rapid change in carrier concentrations at the interface indicates the formation of a depletion region and the charge-transfer equilibrium. The observed carrier density discontinuity confirms the efficient charge transfer and supports the type-II band alignment observed in the energy band diagram. Electrons and holes accumulate in the Mo_2_TiC_2_ and CsSnI_3_ layers, supporting spatial separation of the photogenerated carriers, a crucial step for photodetection applications. Such carrier confinement reduces the interfacial recombination losses and increases the lifetime of the carriers and the charge collection efficiency. As a result, the heterojunction provides superior photocurrent generation, high responsivity, high specific detectivity, and reduced dark current contribution, which are crucial for the optimal performance of self-powered photodetectors. The results of optical and carrier transport investigations demonstrated that the MPH exhibits high visible-light absorption, high photogeneration efficiency, low optical transmission loss, and excellent carrier separation. These synergistic properties provide the basis for the proposed self-powered photodetector to exhibit a high photocurrent, low recombination losses, and superior performance in photodetection applications. The optical calculations in this work were done with the wavelength-independent material parameters adopted in the device model. The optical response was measured mainly in the spectral range of 300–900 nm, which covers the upper range of the spectral region around the reference band edges of CsSnI_3_ (1.30 eV, corresponding to ~954 nm) and Mo_2_TiC_2_ (1.35 eV, corresponding to ~918 nm). Furthermore, the calculated response outside the selected spectral window is very small as demonstrated in Figure 3a–d. Hence, the key operating spectral range was chosen to be 300–900 nm for checking the response of the photodetector.

## 5. Thickness and Spectrum Optimization

Figure 4a presents the external quantum efficiency (EQE) as a function of wavelength for different CsSnI_3_ perovskite thicknesses. The EQE is the ratio between collected charge carriers and absorbed photons [41]. It gradually rises in the visible region and peaks at 80–84% in the range 700–850 nm before decreasing rapidly toward the absorption edge. Among the investigated thicknesses, the 500 nm thick perovskite layer exhibits balanced performance over the entire spectral range. Increasing the absorber thickness of the photodiode from 400 to 500 nm increases the photon absorption and the photocarrier generation, leading to an increase in the EQE of the photodetector. However, an increase to 600 nm induces only a slight improvement and can even increase carrier transit distance and recombination probability. Therefore, the value of 500 nm was found to be the optimum value for subsequent simulations. The EQE spectra at various Mo_2_TiC_2_ thicknesses are presented in Figure 4b.

We observed that the EQE drops sharply with increasing thickness of Mo_2_TiC_2_ from 150 nm to 250 nm and then increases up to 350 nm. Due to the intermediate EQE at 350 nm, we identified the 350 nm thickness as the optimum and used it for further simulations. Furthermore, we reviewed the relevant literature [24,42] and found that the layer of the MXene used for electronic/optoelectronic device applications is generally 50–120 nm, where the MXene layer is considered as a carrier-transport layer with a conductive function. This thickness window is consistent with the decreasing EQE that we have observed for thicker Mo2TiC_2_ layers in our simulations. The selected 350 thickness may be more of a practical thickness range for the device. This thickness of the MXene film is assumed as a homogeneous layer in the modeling on the basis of a moderate EQE result, and there are several possibilities for the anisotropic conductivity, inter-flake transport, and thickness-dependent optical absorption of stacked MXene films that were not explicitly considered in the model. The charge carrier generation and recombination of the optimized MPH-PD are presented in Figure 4c. The generation rate is consistently much higher than the recombination rate in the active zone, indicating that the incident photons are being used efficiently and the losses due to recombination are low. The considerable difference at the heterointerface is related to the extent of charge transfer and carrier separation. The distribution of the built-in electric field along the length of the device is presented in Figure 4d. The electric-field peak at the MPH interface is quite sharp, reaching a maximum value of 3.5 × 10^5^ Vcm^−1^, indicating the formation of a strong depletion region resulting from the band bending and charge redistribution at the type-II heterojunction. This behavior is desirable for self-powered photodetectors as it enables efficient capture of the carriers at zero bias. In order to investigate the impact of the incident solar spectrum, we calculated the I–V curve under different solar spectra, as shown in Figure 5a. The optimized MPH-PD I–V characteristics are presented in Figure 6a under monochromatic light illumination ranging from 300 nm to 700 nm and a typical AM1.5G 1 sun solar spectrum. As the incident wavelength varies over the above-mentioned range, the photocurrent is evidently wavelength-dependent throughout the range of bias studied. The AM 1.5G spectrum contains the most photons, which results in the highest photocurrent. The photocurrent starts to increase slowly when the wavelength increases from 300 nm to 700 nm. The photocurrent is considerably higher under forward bias than under reverse bias because of the better carrier injection and transport across the heterojunction. The higher photocurrent at longer wavelengths is due to the effective charge extraction of the MXene layer and the high optical absorption of the CsSnI_3_ absorber. The results confirm the proposed device’s broadband photoresponse over the visible spectrum. The enhancement of the photocurrent under AM 1.5G illumination indicates the ability of the type-II heterojunction to collect the photocarriers efficiently and maximize the use of the incident sunlight. In order to investigate the impact of the thickness of the absorber layer on performance parameters, we calculated the key performance parameters—responsivity, detectivity, and noise equivalent power (NEP)—of the MPH-PD at three different absorber layer thicknesses (400, 500, and 600 nm), as shown in Figure 5b–d. The spectral responsivity presented in Figure 5b demonstrates a clear peak in the visible spectrum, reaching a peak of roughly 0.38–0.40 A/W around 500–600 nm, depending on the thickness of the absorber. The responsivity is defined by Equation (4) [43,44]:

(4) $$R=\frac{q\lambda (EQE)}{hc}$$

where q is the elementary charge (1.6 × 10^−19^ C), λ is the wavelength (550 nm), h is Planck’s constant, and c is the speed of light. Although responsivity and detectivity are highest for 600 nm, the performance enhancement above the 500 nm configuration is marginal. The performance improvement with the thickness of the absorber is attributed to the greater absorption of photons before transmission losses occur, which in turn increases the number of electron–hole pairs generated. However, outside the optimum thickness range, a higher recombination of carriers can occur. Due to material consumption, fabrication complexity, and carrier transport limitations of thicker absorbers, a thickness of 500 nm for the CsSnI_3_ layer was chosen as the optimum thickness for the proposed photodetector. The maximum responsivity in the visible range is consistent with the intrinsic bandgap of CsSnI_3_ of 1.3 eV, which results in considerable absorption in the range of 400–800 nm. The observed decrease in responsivity beyond 550 nm is due to the reduction in the energy of the photons at longer wavelengths approaching the absorption edge, resulting in a decrease in the efficiency of photogeneration. Figure 5b presents the variation in specific detectivity (D*) defined by Equation (5) [44,45], with the wavelength of the incident light spectrum at different thicknesses of the absorber layer:

(5) $$D^{*}=\frac{R\sqrt{A}}{\sqrt{2qI_{d}}}$$

where R is the responsivity, A is the standard surface area (1 cm^2^), q is the elementary charge, and I_d_ is the dark current (~10^−14^ mA/cm^2^ at zero bias) for the same effective illuminated area under standard AM1.5G 1 sun illumination. It follows a similar trend as responsivity, with a maximum of roughly 2.12 × 10^15^ Jones (where Jones is cm·Hz^1/2^ W^−1^) in the visible spectrum. The enhancement of detectivity is attributed to two main factors: an increase in photocurrent in the presence of illumination and a decrease in the dark current under reverse bias conditions. The MXene layer is particularly important for improving performance, as it provides efficient paths for extracting carriers and reduces recombination losses at the interface. The sensitivity of the system was determined with noise-equivalent power (NEP), which is the optical power required per square root of bandwidth to generate a photocurrent equal to the noise contribution. NEP can be determined as illustrated in Equation (6) [44,46]:

(6) $$NEP=\frac{\sqrt{A}}{D^{*}}=\frac{\sqrt{2qJ_{d}}}{R}\;$$

The NEP exhibits an inverse behavior with detectivity, with a minimum value of roughly 1–2 × 10^−16^ WHz^−1/2^ in the visible region; thereafter, it increases significantly at shorter and longer wavelengths. The minimum NEP is obtained in the region of wavelengths at which the absorption and carrier generation are maximum, which further confirms the best sensitivity of the device in the visible spectrum. The 400–600 nm absorbers exhibit an almost identical NEP among the different thicknesses studied, indicating better photodetection performance. This behavior is due to the improved responsivity of the system and the reduction in noise contributions from the heterojunction due to effective carrier separation.

## 6. I–V Characteristics and Thermal Stability

The I–V curve under the standard solar spectrum (AM1.5G 1 sun) exhibits typical behavior in the dark and a significant photoresponse under illumination at zero bias, as shown in Figure 6a. The dark current has a value of ~10^−12^–10^−14^ mAcm^−2^ at zero bias, which is very low, indicating a high-quality junction of MPH and a very low thermally generated current in the dark state. MPH exhibits an increase in photocurrent by several orders of magnitude when it is illuminated, reaching current densities of roughly 1–10 mA/cm^2^ in the reverse bias region. This high light-to-dark current ratio (10^14^ at 0 V) confirms the effective generation and extraction of the photocarriers, which is due to the built-in electric field at the heterojunction and the high electrical conductivity of the MXene layer. The built-in potential at the interface is crucial in order to reduce the dark current and to facilitate directional carrier transport under illumination, thus improving the signal-to-noise ratio and detectivity. We further tested the device at Vbias = 0 V and found a short-circuit current density (Jsc) of 38 mA cm^−2^ and an open-circuit voltage (Voc) of 0.58 V, enabling the device to provide more appropriate zero-bias metrics. To investigate the thermal stability of the system, we studied the responsivity (R), specific detectivity (D) and noise equivalent power (NEP) with operating temperature as presented in Figure 6b. The responsivity and detectivity increase with temperature and then decrease at higher temperatures, with peaks of 0.37 AW^−1^ and 6.3 × 10^15^ Jones near 300 K, respectively. The NEP increases with both decreasing and increasing temperatures and has a minimum value of approximately 1.6 × 10^−16^ WHz^−1/2^ at 300 K. The phenomenon is observed due to the competing effects of carrier transport and thermal recombination. The carrier collection efficiency is reduced at low temperatures due to the limited carrier mobility and the reduced temperature activation [47]. As the temperature approaches room temperature, the photocarrier extraction improves with the enhancement of carrier transport, which also increases the responsivity and detectivity. However, at higher temperatures, thermally induced recombination and leakage currents become more significant, reducing detector sensitivity and increasing noise [48]. The optimum photodetector performance is achieved under ambient operating conditions, as shown by the simultaneous achievement of maximum responsivity and detectivity and minimum NEP near 300 K. The device shows consistent performance over the investigated temperature range. The present temperature analysis is a numerical evaluation of the temperature dependence of the performance, and is not an actual thermal-aging or cycling experiment to assess the long-term excellent thermal stability. It needs an in-depth analysis of semiconductor and junction parameters such as band gap, carrier mobility, intrinsic carrier concentration, carrier lifetime, recombination characteristics, band offsets and contact barriers relative to temperature.

The moderate change in capacitances and the unchanged impedance properties in the temperature range from 260 to 360 K (Figure 7) suggest that the electrical response of the MXene/CsSnI_3_ heterojunction does not change significantly in the investigated temperature range.

The C-f, Nyquist impedance and G-f characteristics evaluated between 260 and 360 K are shown in Figure 7, which is particularly helpful for electrical RC/relaxation analysis. The MXene/CsSnI_3_ junction capacitance is nearly frequency-independent, ranging from 1 Hz to 10^8^ Hz, and the values of approximately 17.49–18.00 nF cm^−2^ are obtained over a range of 260–360 K, as shown in Figure 7a. This low-frequency dispersion suggests that the simulated heterojunction is not found to have a significant capacitive relaxation in the range of frequencies studied. The relatively small capacitance change also suggests that the electric response of the junction is fairly temperature independent within the range of temperatures investigated.

The Nyquist plots show the characteristic depressed semicircular response with the real component of the impedance ranging from about 230–250 Ω·cm^2^ as shown in Figure 7b. The response in terms of real impedance is increasing gradually with temperature, which suggests a temperature-dependent resistive/relaxation contribution. The approximate agreement between the low-frequency conductance and the inverse of the characteristic impedance (G∼1/R) further supports the consistency of the electrical response.

The conductance is almost constant at low and intermediate frequencies, 4.0–4.4 × 10^−3^ S·cm^−2^, and rises significantly at frequencies close to 10^8^ Hz as can be seen in Figure 7c. The high-frequency increase suggests that the AC carrier response/relaxation process starts to become faster. Although direct transient rise/fall time calculation is not included in the present steady-state device model, these analyses can be useful for a quantitative estimation of RC characteristic time/frequency. The extracted characteristic resistance and capacitance result in an estimated RC time constant of ~4.0–4.5 μs and a related RC characteristic frequency (f_RC_ = 1/2πfC) of ~35–40 KHz. The responsivity of the proposed Mo_2_TiC_2_/CsSnI_3_ device is 0.38 A/W and the detectivity is 2.12 × 10^15^ Jones at 550 nm. The responsivity is close to the reported heterojunction photodetectors and the detectivity is higher than that of most of the devices listed in Table 2. Importantly, the proposed device offers a competitive responsivity and a high detectivity under simulated conditions, which indicates its high-performance applications in the field of photodetection.

The proposed Mo_2_TiC_2_/CsSnI_3_ heterojunction photodetector exhibits promising optoelectronic properties, competing with the performance of previously reported heterojunction photodetectors as described in Table 2. It is essential to acknowledge that only numerical simulations using COMSOL Multiphysics were used for the evaluation of device performance under idealized conditions. The calculated specific detectivity is an upper bound (shot-noise limited), because the present model assumes only the noise from the simulated dark current. In real devices, other sources such as Johnson, generation–recombination, 1/f and contact/interface noise can add to the overall noise, thereby affecting the experimentally attainable value of the detectivity. It was assumed that the material properties, the quality of the interfaces, and the distributions of the defects are spatially uniform in this analysis. However, defects such as interface roughness, grain boundaries, surface oxidation of MXene, and non-uniform distributions of defects in the structural properties of these devices can provide additional recombination channels, which may reduce the carrier lifetime, responsivity, and detectivity. Furthermore, the present model neglects the effects of contact resistance, interfacial trap-assisted tunneling, and carrier transport anisotropy. These effects can play important roles in nanoscale heterostructures and affect the real device response.

In future studies, researchers should focus on experimental fabrication and characterization of the proposed heterojunction to verify the simulation results. Incorporation of realistic interface states, contact effects, and defect-assisted transport modeling will provide a comprehensive understanding of carrier dynamics. Surface passivation, defect engineering, and compositional tuning of the surface terminations on MXene (-O, -OH, and -F) may enable improved carrier extraction and reduce the recombination loss. In addition, the study of plasmonic nanostructures, optical resonators, and multi-dimensional architectures of MXene/perovskites can improve light–matter interactions and expand the spectral response range toward the near-infrared region. Lastly, transient photoresponse analysis, performance analysis for various frequencies, and reliability tests under different environmental conditions should be performed to determine the suitability of the device for practical applications in broadband and self-powered photodetection.

## 7. Conclusions

In this work, a novel Mo_2_TiC_2_/CsSnI_3_ staggered (type-II) heterojunction photodetector (MPSH-PD) was designed and studied using COMSOL Multiphysics. An extensive optoelectronic simulation study was performed to optimize the proposed FTO/Mo_2_TiC_2_/CsSnI_3_/Au architecture to elucidate the role of heterojunction band alignment, carrier dynamics, optical absorption, layer thickness, and operating temperature in the performance of the proposed photodetector. The determined conduction-band and valence-band offsets of 0.30 eV and 0.35 eV, respectively, confirm the formation of a type-II heterojunction, which not only ensures efficient charge separation but also reduces carrier recombination and facilitates carrier extraction through the highly conductive MXene layer. The device exhibited strong visible-light absorption, an internal quantum efficiency exceeding 80%, enhanced photogeneration efficiencies, and a built-in electric field at the Mo_2_TiC_2_/CsSnI_3_ interface. The thickness optimization revealed that a 500 nm CsSnI_3_ absorber layer and a 350 nm Mo_2_TiC_2_ MXene layer possess the optimum thickness balance between photon harvesting and carrier transport. The optimized MPSH-PD exhibited a very high light-to-dark current ratio of 10^8^ under reverse bias and 10^13^ at zero bias under AM 1.5G illumination, demonstrating its superior self-powered photodetection ability. Moreover, a responsivity of 0.38 AW^−1^, a peak detectivity of 2.12 × 10^15^ Jones, and a minimum noise-equivalent power of 4.7 × 10^−16^ W Hz^−1^ at an incident wavelength of 550 nm were observed. Analysis based on temperature further demonstrated the optimal performance of photodetection near room temperature, highlighting the robustness of the proposed device in terms of its operational temperature. Thus, the improved optoelectronic characteristics achieved by the synergic combination of Mo_2_TiC_2_ MXene and lead-free CsSnI_3_ perovskite demonstrate the effectiveness of type-II heterojunction engineering in high-performance photodetectors. The achieved results demonstrate that the proposed MPSH-PD is a promising platform for the realization of environmentally friendly, highly sensitive, and self-powered photodetection technologies. In addition, it provides useful design principles for further experimental validation and development of advanced optoelectronic sensor applications.

## Figures and Tables

**Figure 1:**
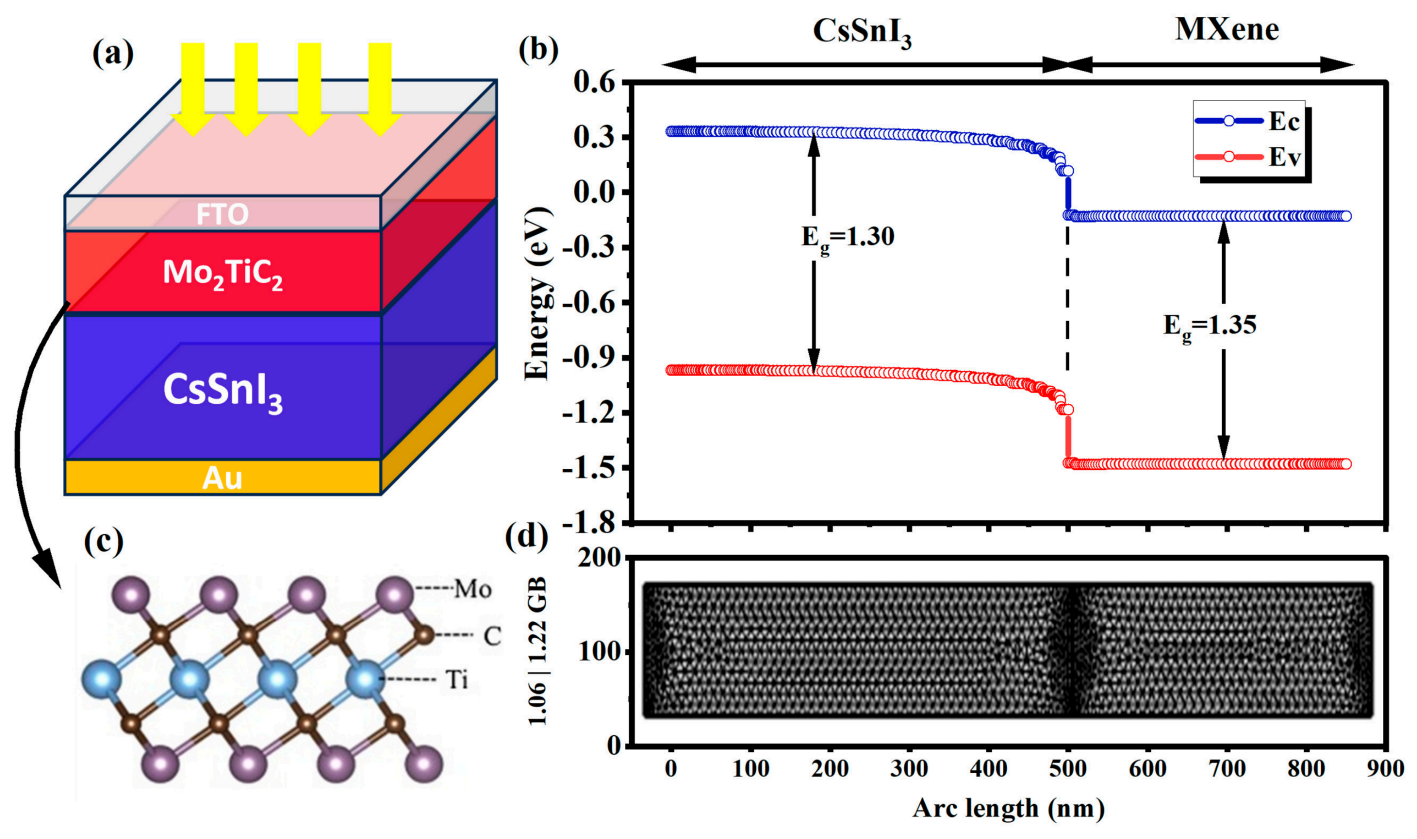
(**a**) Architecture of the proposed MPH-PD, (**b**) energy band diagram, (**c**) Mo_2_TiC_2_ structure, and (**d**) finite-element mesh used for the simulations.

**Figure 2:**
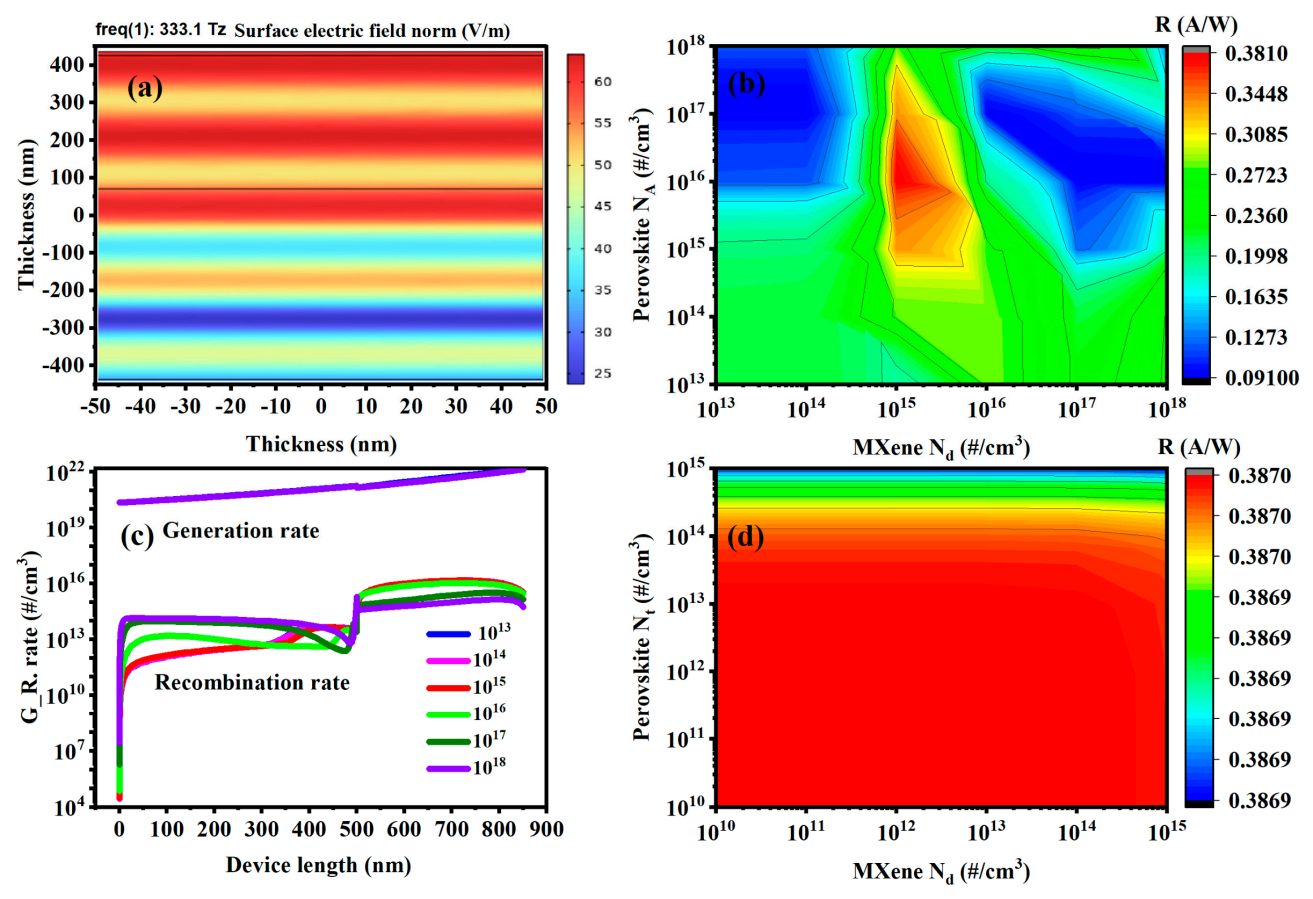
(**a**) The spatial distribution of the electric field norm at the surface of the MPH-PD. Along the vertical direction, from bottom to top, are the Au electrode, CsSnI_3_, Mo_2_TiC_2_, and FTO, (**c**) generation and recombination rate at different carrier densities of CsSnI_3_, and (**b**,**d**) responsivity at different doping densities of the CsSnI_3_ and MXene layer.

**Figure 3:**
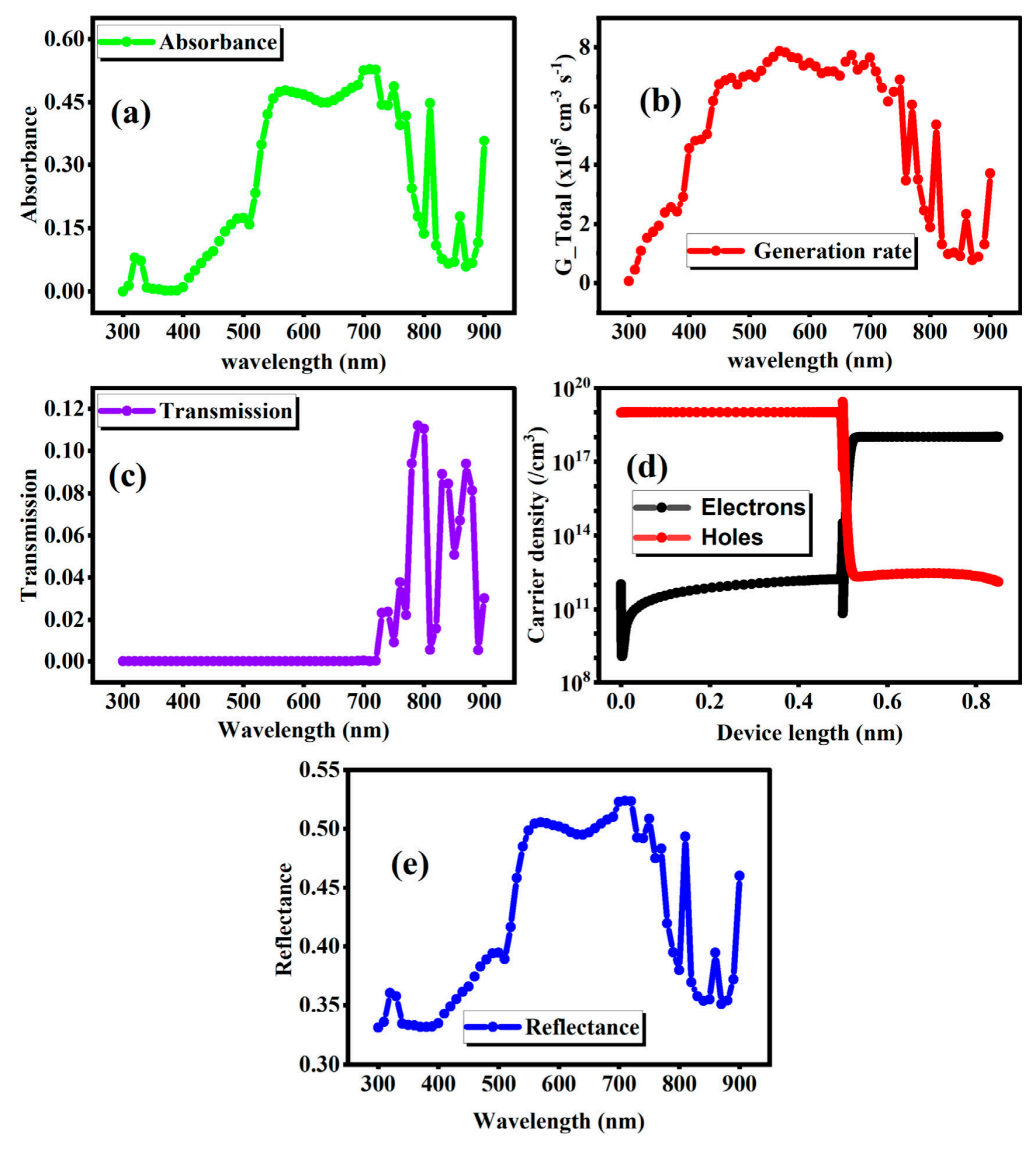
Optical Properties: (**a**) absorbance, (**b**) photogeneration rate, (**c**) transmittance, and (**d**) distribution of the carrier density of the proposed MPH-PD, (**e**) reflectance.

**Figure 4:**
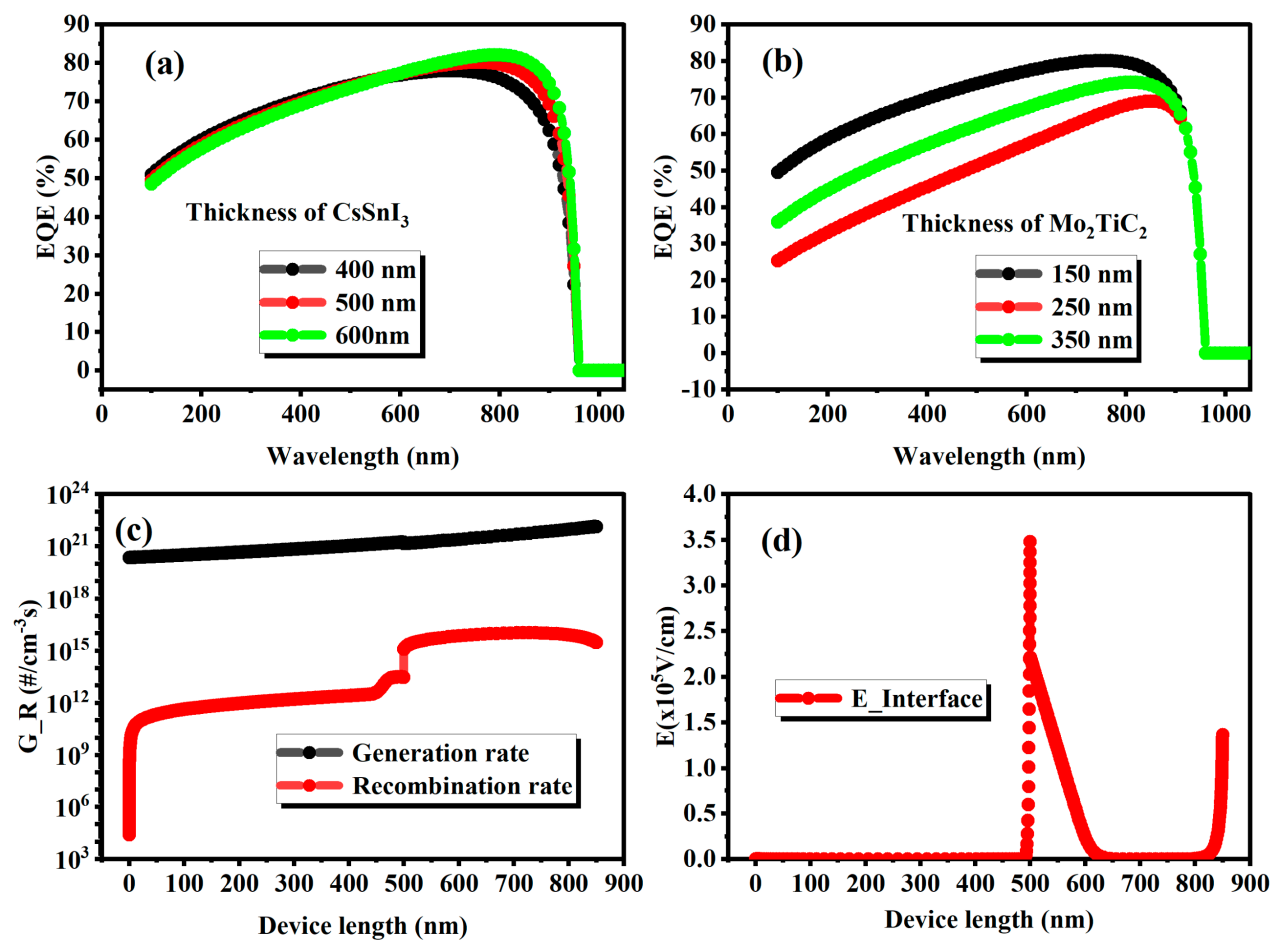
External quantum efficiency (EQE) as a function of wavelength for different thicknesses of (**a**) CsSnI_3_ and (**b**) Mo_2_TiC_2_, (**c**) charge carrier generation and recombination rates, and (**d**) built-in electric field along the length of the device.

**Figure 5:**
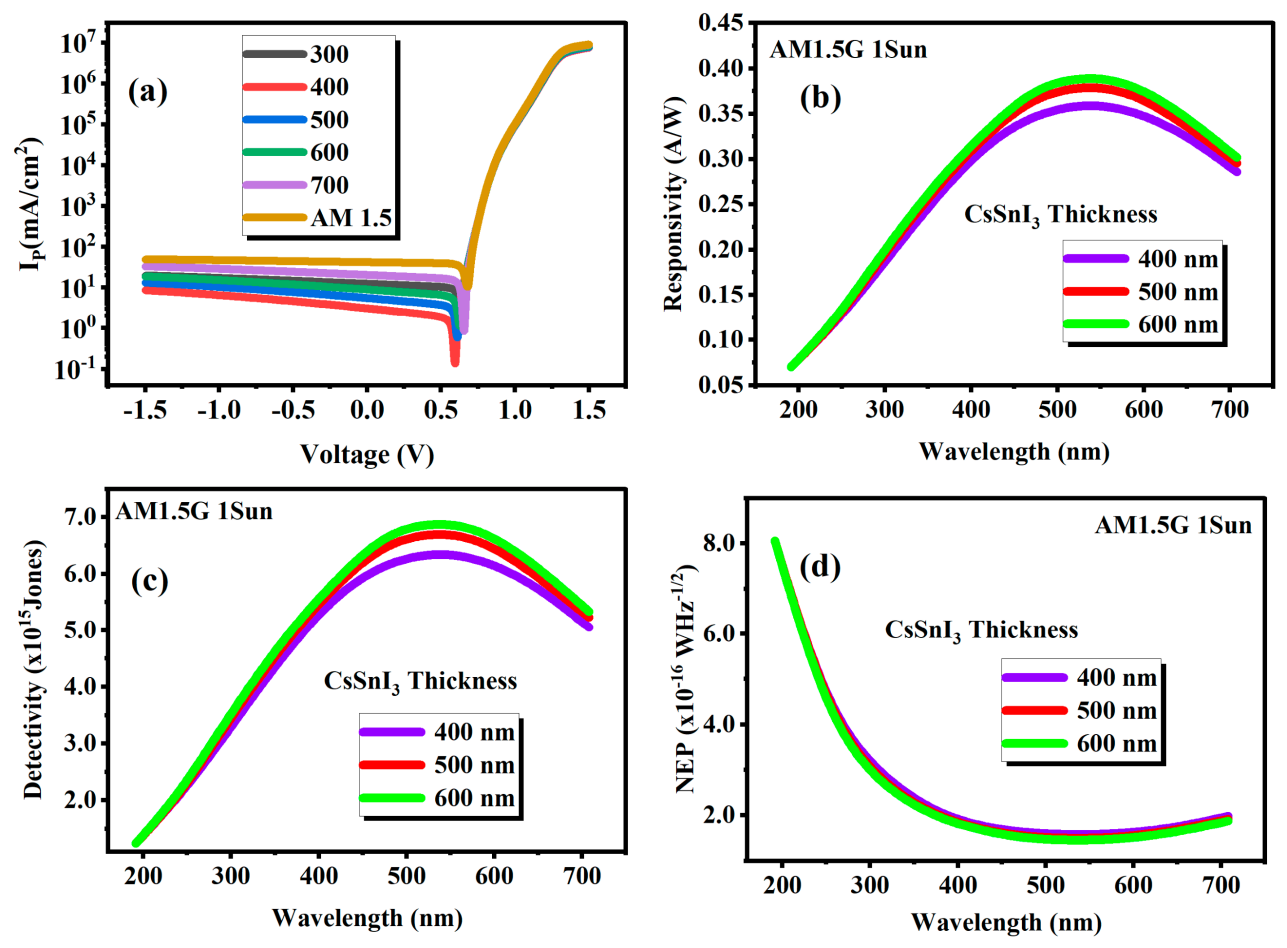
(**a**) I–V curve at different wavelengths of the solar spectrum and key performance parameters, (**b**) responsivity, (**c**) detectivity, and (**d**) noise-equivalent power (NEP) for three different absorber-layer thicknesses.

**Figure 6:**
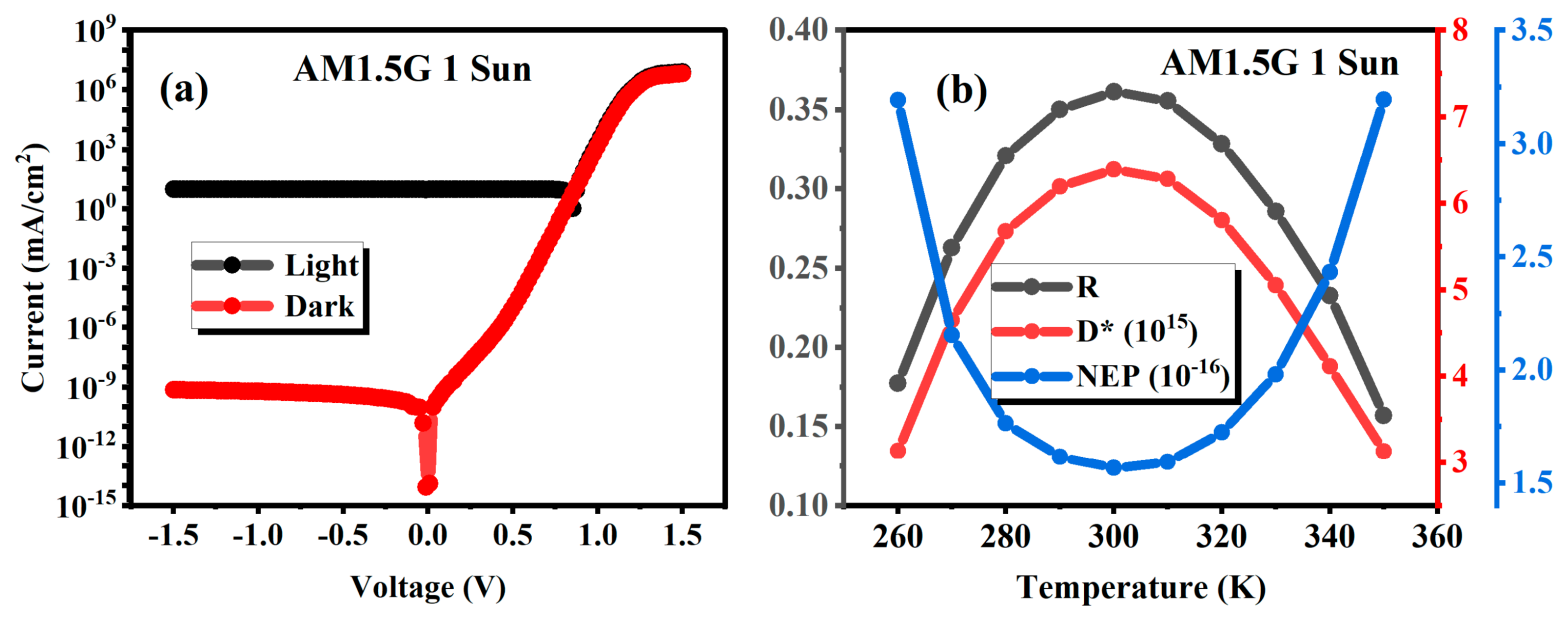
(**a**) Typical behavior of MPSH-PD in the dark and under illumination and (**b**) the effect of temperature on key performance parameters.

**Figure 7:**
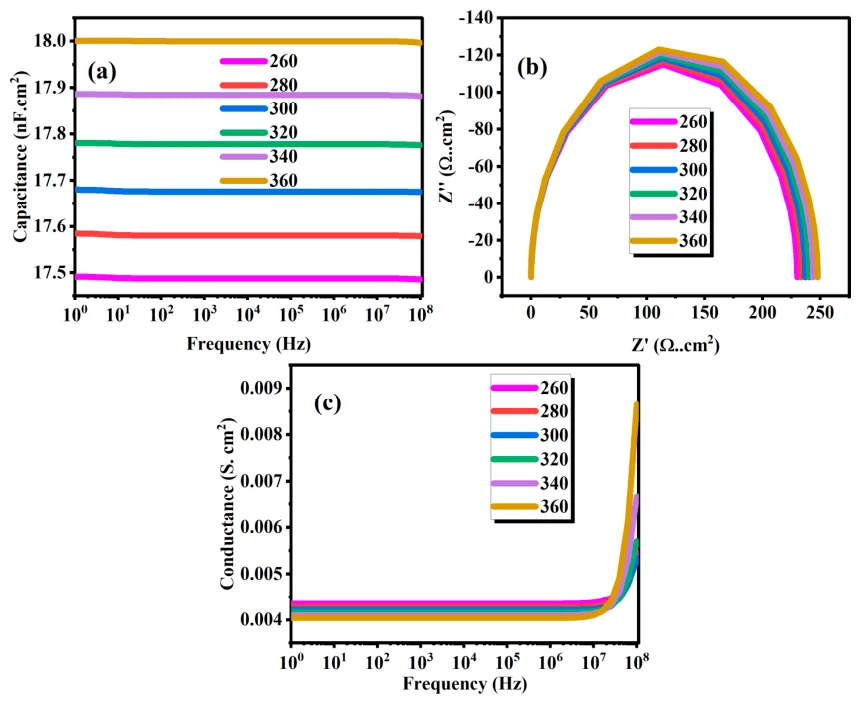
Electrical RC/relaxation analysis (**a**) C-f characteristics (**b**) Nyquist plot (**c**) G-f characteristics evaluated from 260 to 360 K.

**Table 2:** Comparison of the performance of the MPSH-PD with previously reported heterojunction photodetectors.

Architecture	Wavelength (nm)	Method	Responsivity (A/W)	Detectivity (Jones)
FTO/MoS_2_/Au [49]	700	Simulation	0.33	1.17 × 10^14^
Graphene/GaAs [50]	725	Experimental	0.51	1.16 × 10^11^
PbS/TiS_3_ [51]	700	Simulation	0.36	3.90 × 10^13^
CsSnI_3_ NWs PD [52]	-	Experimental	0.24	1.18 × 10^12^
CsSnI_3_/MoS_2_ [25]	500	Simulation	0.59	4.25 × 10^13^
Mo_2_TiC_2_/InP [20]	1550	Simulation	2.31	2.10 × 10^13^
Mo_2_TiC_2_/CsSnI_3_	550	Simulation	0.38	2.12 × 10^15^

## Data Availability

The original contributions presented in this study are included in the article. Further inquiries can be directed to the corresponding author.
